# Applying negative ions and an electric field to countermeasure droplets/aerosol transmission without hindering communication

**DOI:** 10.1038/s41598-023-40303-5

**Published:** 2023-08-26

**Authors:** Kaito Kanda, Hisaaki Nishimura, Takuya Koiso, Kousuke Takemoto, Kazuma Nakagoe, Tetsuya Yamada, Masaharu Takahashi, Mariko Hanafusa, Tomoki Kawahara, Yasuko Yanagida, Jin Kuramochi, Takeo Fujiwara

**Affiliations:** 1 Laboratory for Future, Interdisciplinary Research of Science and Technology, Tokyo Institute of Technology, Kanagawa, Japan; 2 Department of Global Health Promotion, Tokyo Medical and Dental University, Tokyo, Japan; 3 Department of Aeronautics and Astronautics, The University of Tokyo, Tokyo, Japan; 4 Research Center for Frontier Medical Engineering, Chiba University, Chiba, Japan; 5 Kuramochi Clinic Interpark, Utsunomiya, Japan

**Keywords:** Infection, Biomedical engineering, Electrical and electronic engineering, Applied physics

## Abstract

In the COVID-19 pandemic, lockdown and acryl partitions were adopted as countermeasures against droplets/aerosol infections; however, these countermeasures restrict communication. In this study, a blocking device was developed using negative ions and an electric field. The device blocks mists simulating droplets/aerosol by a maximum of 89% but transmits light and sound, which is important for communication. The device demonstrated effective blocking performance for aerosol, including the COVID-19 virus spread from patients in a clinic. Our device can help prevent infections without disrupting communication.

## Introduction

Infections are spread via droplets/aerosol produced by talking or coughing. For example, H1N1 influenza, severe acute respiratory syndrome (SARS), and Middle East respiratory syndrome (MERS) have spread due to droplets/aerosol^1^. Additionally, COVID-19 is mainly transmitted through droplets/aerosol and contact^2^, leading to a serious global pandemic. Therefore, countermeasures against droplets/aerosol transmission are essential for maintaining public health.

In the early stages of the COVID-19 pandemic, lockdowns were applied as strict countermeasures worldwide^3^. However, they were unsustainable countermeasures because of severe problems including reduced face-to-face interaction and induced severe economic losses^4^, mental illness such as depression^5,6^, and, as we have previously reported, stunted social-emotional skills among preschool children and increased abusive parental behaviors^7,8^. Therefore, sustainable countermeasures against pandemics without disrupting economic activities and daily interactions are required.

Partitions are countermeasures used to block droplets/aerosol transmission during communication. The disadvantages of the partitions are the reflecting and blocking of sound and light. The reflected voice annoys the speaker^9^, and the blocking effect disturbs communication. The reflected light prevents the reading of facial expressions. Therefore, a method that transmits sound and light while blocking droplets/aerosol is required.

To achieve sustainable countermeasures against infections without impairing communication, we focused on the use of negative ions and an electric field, which block droplets/aerosol but transmit sound and light. Negative ions and an electric field are conventionally applied in air cleaners because they efficiently collect airborne viruses and bacteria^10–12^. We considered that negative ions and electric field are solutions applicable for blocking droplets/aerosol at places where people communicate.

In this study, we propose a device that blocks droplets/aerosol using negative ions and an electric field. Different blocking devices with heights between 8 and 50 cm were prepared, and an electric field was simulated. The effects of negative ions and the electric field on floating objects in the atmosphere, effect of the electric field in guiding the negative ions, blocking performance of the blocking device, blocking performance at different height-positions, and transmission and reflection of light and sound were observed. The blocking device was finally investigated for blocking against aerosol containing COVID-19 viruses.

## Results

### Fabrication of the blocking device

**Figure 1:**
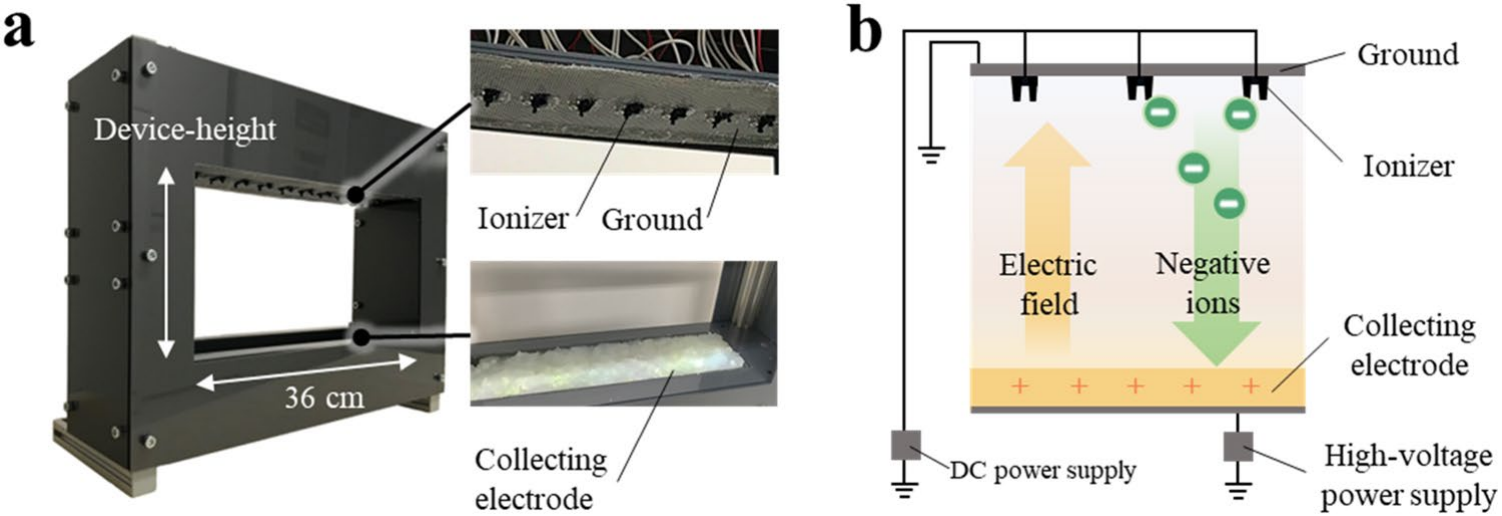
(**a**) Picture of the blocking device. (**b**) Schematic of the device.

The blocking device (shown in Fig. 1a) comprises three main parts: an ionizer, ground, and collecting electrode. Blocking device-width is 36 cm, and the height was changed for each experiment. Figure 1b shows the schematics of the device. Three to eleven ionizers (Huizhou Pengkui Technology Co., China) were attached at equal intervals on the centerline of the top surface of the blocking device. The ionizers were connected to a 12 V DC power supply (PR18-3A, TEXIO). Negative ions were generated via corona discharge at two electrodes of the ionizer. Notably, ozone was not detected using a semiconductor sensor (Ozon checker OC-300, Ozon Technica CO., LTD) during the generation of negative ions in the ionizer. The detection limit of the sensor was 0.001 ppm. A metal mesh, as a ground, was attached to the top surface of the blocking device surrounding the ionizers. A collecting electrode was attached to the bottom of the blocking device. A total voltage of + 15 kV was applied to the collecting electrode using a high-voltage power supply (GS30P, Green Techno).

### Electric field simulation

**Figure 2:**
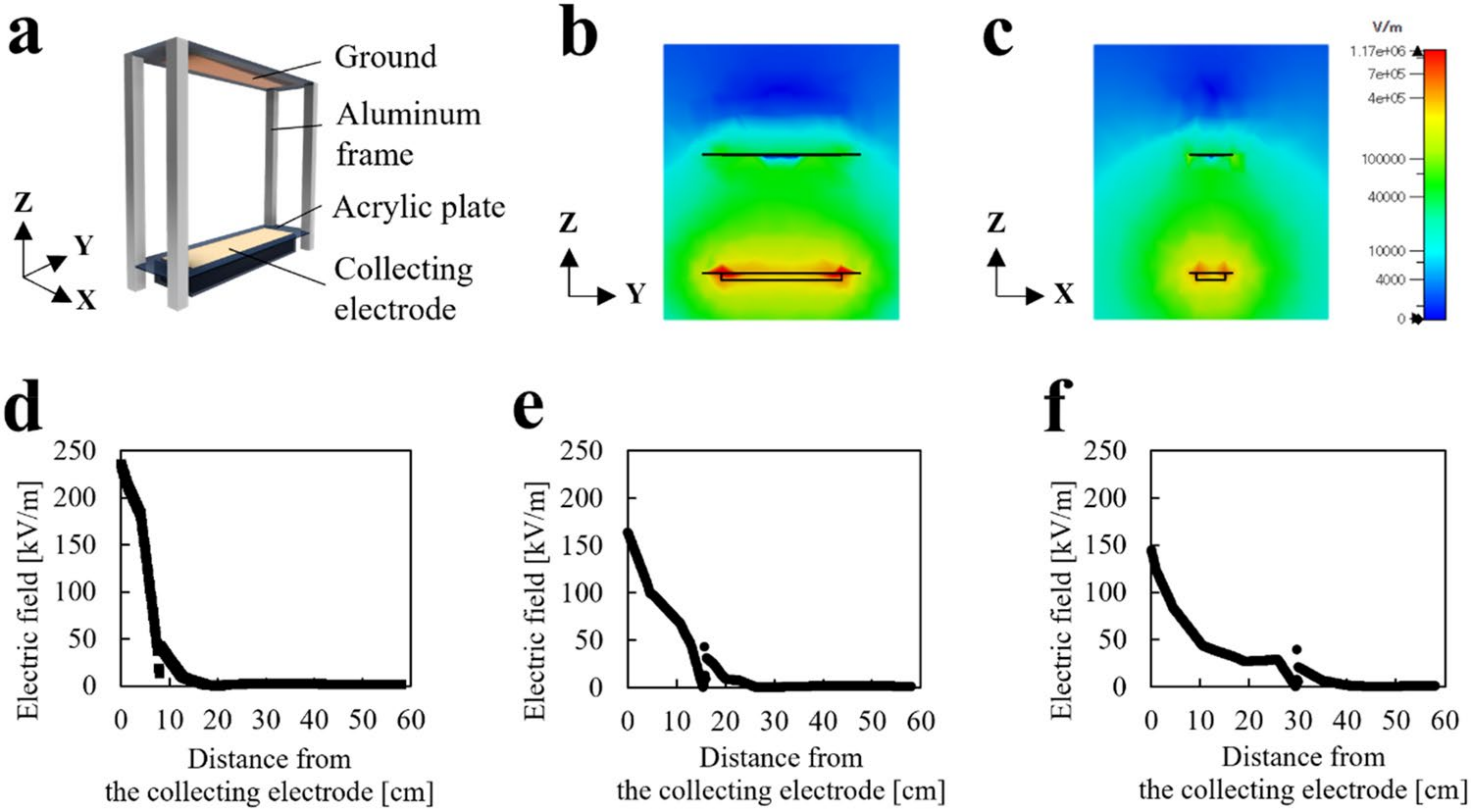
(**a)** Simulation model. (**b**,**c**) Simulation result of the device with a height of 30 cm. (**d**–**f**) Electric field intensity at the distance from the collecting electrode surface. The device-height was 8, 16, and 30 cm for (**d**), (**e**), and (**f**), respectively.

The electric field was simulated using the model shown in Fig. 2a. A potential of 15 kV is applied to the collecting electrode and 0 kV to the ground electrode. Figure 2b and c show the electric field distribution in Y–Z and X–Z directions for the blocking device with a height of 30 cm. The electric field intensity in the blocking device was almost constant in the Y- and X-directions. Figure 2d–f shows electric field intensity in the Z-direction for blocking device with height of 8, 16 and 30 cm, respectively. The electric field intensity was high around the collecting electrode and the intensity was decreased along with the distance from the collecting electrode (Z-direction); this decrement of electric field can be caused by finite electrode and edge effect. The maximum electric field intensity was 235, 164, and 145 kV/m in the blocking device with heights of 8, 16, and 30 cm, respectively. These simulation results suggest the blocking performance around the collecting electrode is more effective than that around the ground electrode.

### Effect of negative ions and electric field

**Figure 3:**
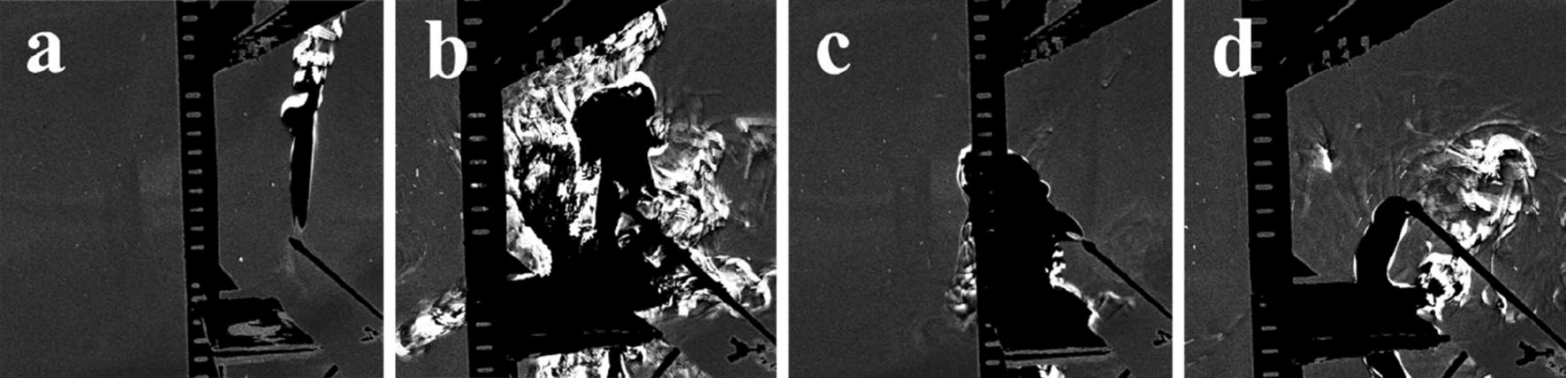
Effect of negative ions and the electric field on the smoke flow. The height between the ionizer and collecting electrode was 22 cm for (**a**) and (**b**) and 15 cm for (**c**) and (**d**). (**a**) Smoke flow without the negative ions and electric field. (**b**) Smoke flow with only the negative ions. (**c**) Smoke flow with only the electric field. (**d**) Smoke flow with the negative ions and electric field.

To investigate the effects of negative ions and electric field on objects flowing in the atmosphere, the smoke flow was observed. Figure 3a–d show pictures captured from video S1, which recorded the smoke flow. When negative ions and an electric field were not generated, the smoke moved upward (Fig. 3a). When only negative ions were generated from the ionizers, the smoke was directed downward rapidly and then rebounded against the collecting electrode (Fig. 3b). An ionic wind was also observed when the ionizers were turned on. In the case of the electric field without negative ions, the smoke partially flowed toward the collecting electrode (Fig. 3c). However, almost all the smoke flowed toward the collecting electrode when both negative ions and the electric field were generated (Fig. 3d). In addition, the rebounding of smoke was improved compared to the case of only negative ions (Fig. 3b and d). Figure 3 indicates that the negative ions and electric field induced the smoke to flow toward the collecting electrode. Moreover, using both negative ions and the electric field enhances smoke flow toward the collecting electrode and prevents rebounding, suggesting that negative ions and the electric field potentially block droplets/aerosol.

### Effect of the electric field to guide the negative ions

**Figure 4:**
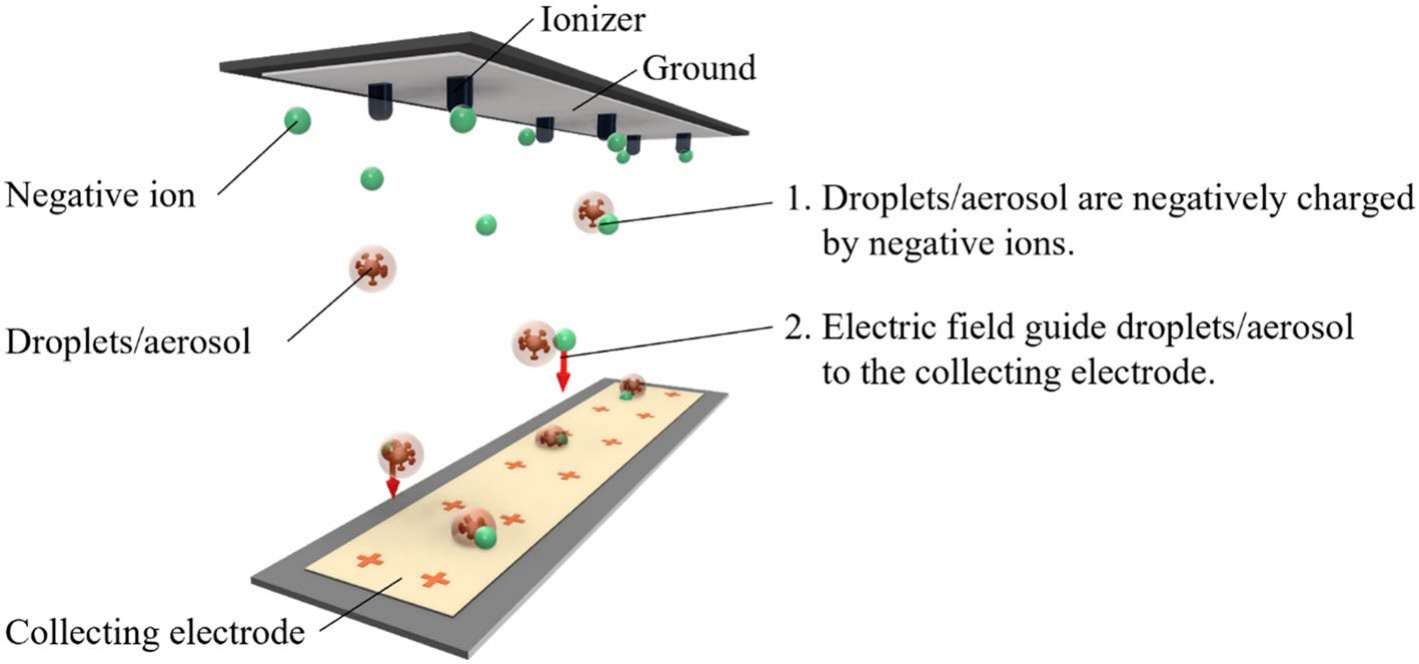
Shematic of the blocking droplets/aerosol.

In the process of blocking droplets/aerosol by the blocking device (Fig. 4), the first droplets/aerosol are negatively charged by the negative ions. The electric field then guides the charged droplets/aerosol to the collecting electrode. Therefore, the performance of the electric field in guiding the negative ions is important. The negative ion concentration distribution around the device was investigated to verify the guiding performance of the electric field.

**Figure 5:**
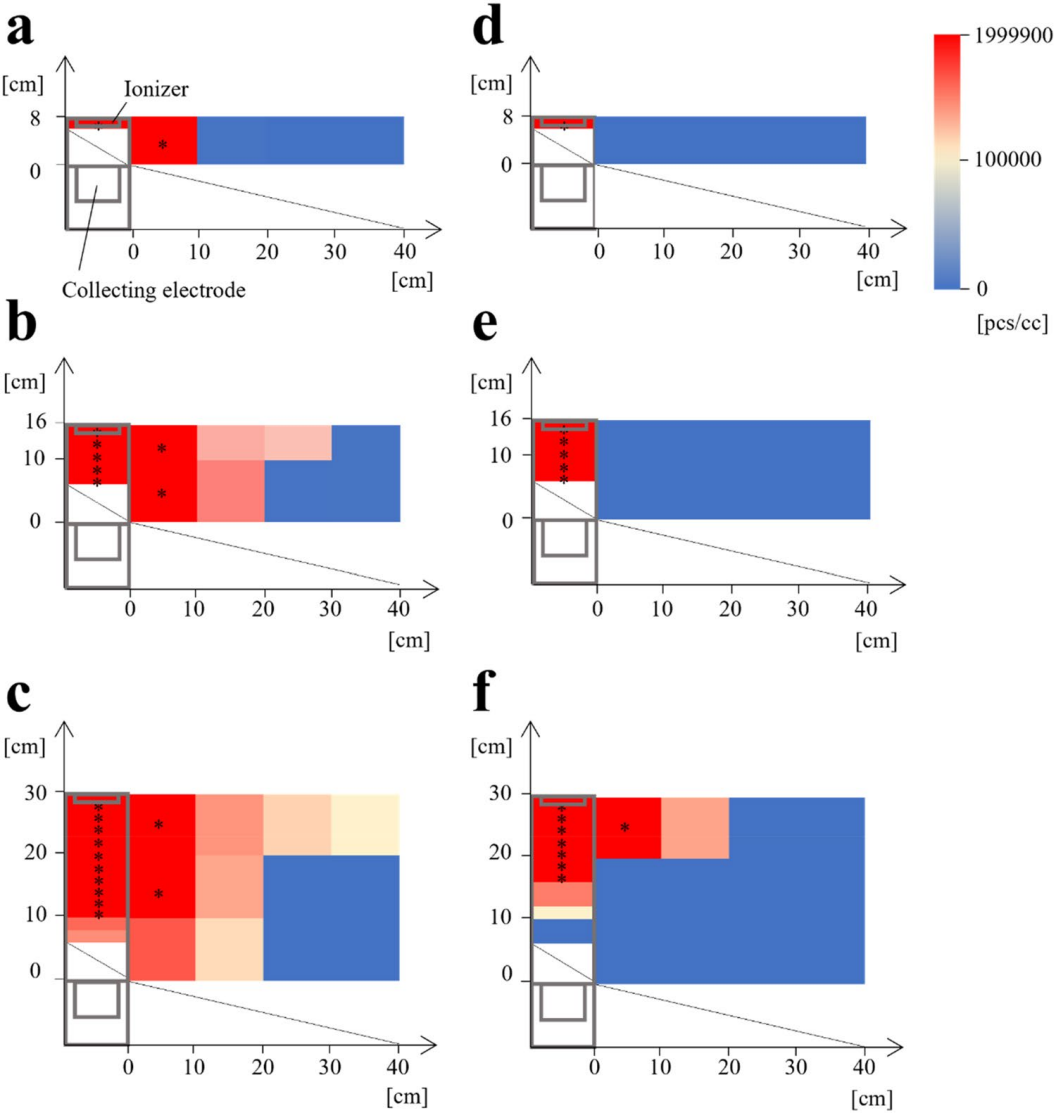
Negative ion concentration distribution around the device. The height of the devices in (**a**) and (**d**) is 8 cm, 16 cm in (**b**) and (**e**), and 30 cm in (**c)** and (**f**). (**a**), (**b**), and (**c**) show the measurement results without the electric field. (**d**), (**e**), and (**f**) show the measurement results with the electric field. Areas marked with * indicate that the negative ion concentration saturated the ion counter equipment’s measurement limitation.

Figure 5a–c show devices with heights of 8, 16, and 30 cm, respectively, without the electric field. High-concentration negative ions were observed inside and outside of the devices, and the negative ion concentration gradually decreased with increasing distance from the device, indicating that the negative ions generated from the ionizers leaked out without the electric field. In particular, more negative ions leaked out using a larger device-height, and the negative ions reached a distance of 40 cm from the blocking devices with a height of 30 cm. Subsequently, Fig. 5d–f show the devices with heights of 8, 16, and 30 cm, respectively, with the electric field. High-concentration negative ions were also observed inside the blocking devices, but the distribution of the negative ions outside of the device drastically decreased because of the electric field effect. Negative ions were not detected using the device with heights of 8 and 16 cm. The device with a height of 30 cm with the electric field released negative ions partially because of the electric field reduction depending on the device-height, as shown in Fig. 3. Note that negative ions were detected at 20 cm from the device (Fig. 5f), which is closer to the device without an electric field (40 cm from the device, Fig. 5c), indicating that the electric field guides the negative ions. The negative ion concentration in the device with a height of 30 cm was decreased (Fig. 5f) compared to that without the electric field (Fig. 5c). This result can be explained by the acceleration of the negative ions by the electric field. The following equation expresses the current $I$ due to negative ions:

1 $$\begin{array}{c}I=Ae{v}_{i}{n}_{i}\end{array}$$

where, $A$, $e$, ${v}_{i}$, and ${n}_{i}$ are the cross-sections of the collecting electrode, elementary charge, negative ion velocity, and negative ion number density, respectively. When charged droplets are accelerated by the electric field, because $I$, $e$, and $A$ are constant, it results in a smaller ${n}_{i}$, as shown in Fig. 5c and f. Figure 5 indicates that the electric field enhances the guiding of negative ions toward the collecting electrode, which is important for blocking charged droplets/aerosol.

### Blocking performance of the blocking device

**Figure 6:**
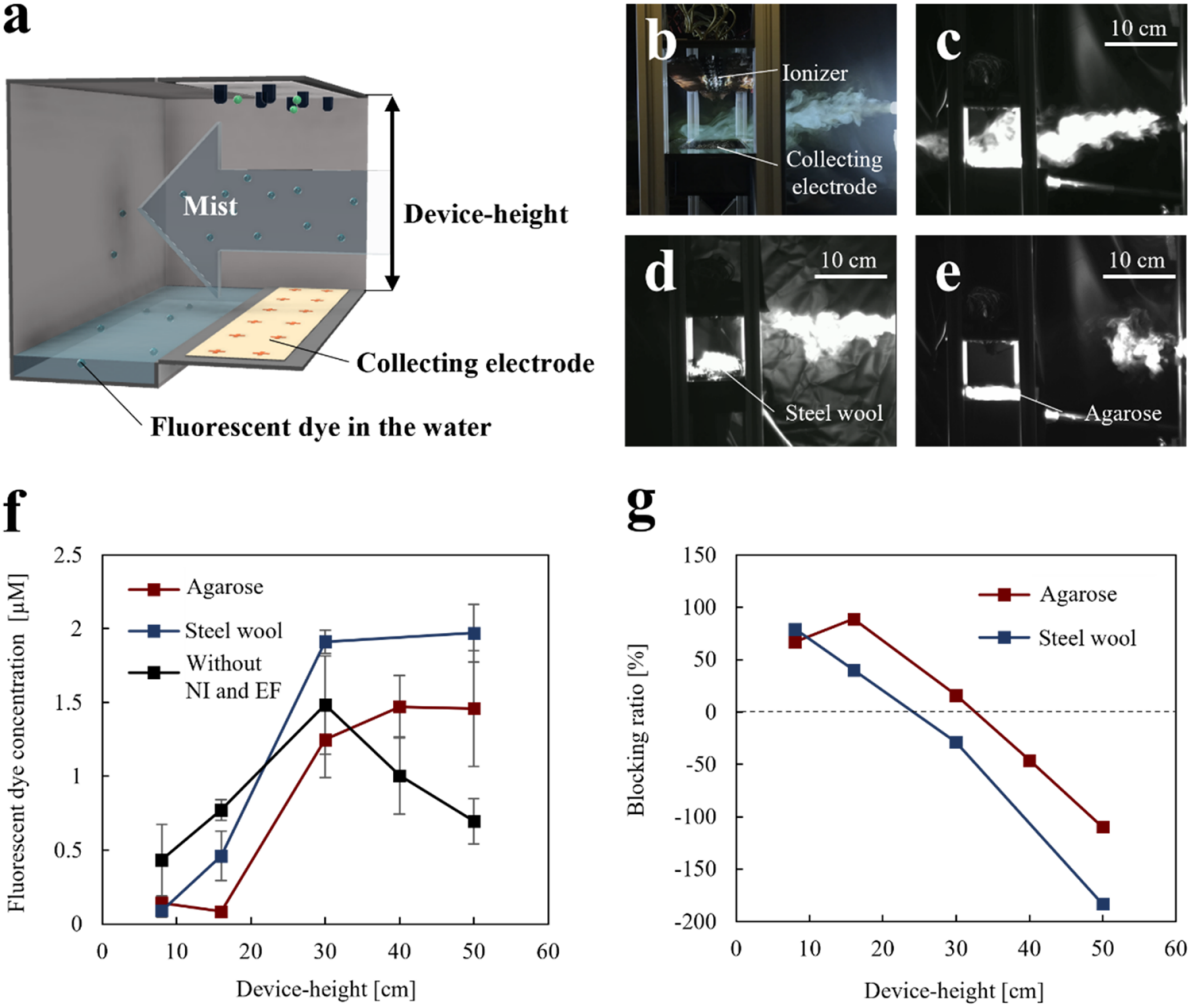
(**a**) Schematic of the experiment setup. (**b**) Representative picture of the blocking device at an 8 cm height using steel wool as the collecting electrode. (**c**) Mist flow without negative ions and an electric field. (**d**) Mist flow with negative ions and an electric field using steel wool as the collecting electrode. (**e**) Mist flow with negative ions and an electric field using agarose as the collecting electrode. (**f**) Relationship between the fluorescent dye concentration and device-height. “Agarose” indicates the device with negative ions and an electric field using agarose as the collecting electrode. “Steel wool” indicates the device with negative ions and an electric field using steel wool as the collecting electrode. “Without NI and EF” indicates the device without negative ions and an electric field. The error bars indicate the deviations of the three experiments. (**g**) Relationship between the blocking ratio and device-height.

The blocking performance of the devices with two types of collecting electrodes was evaluated by changing the blocking device-height (Fig. 6a). The first collecting electrode was agarose-saturated with NaCl to capture particles via surface wettability. The second collecting electrode was steel wool to collect particles using a mesh structure with a large surface area. The height of the blocking device was controlled between 8 and 50 cm. The mist was used to simulate droplets/aerosol generated by a mild cough^13^.

Figure 6b shows a representative picture of the dvice with a height of 8 cm using steel wool as the collecting electrode. The mist flow using the blocking device with “Agarose” or “Steel wool” at different heights is shown in Video S2. Figure 6c–e show captured pictures from Video S2 using a device with steel wool or agarose as a collecting electrode. Figure 6c shows that the mist passes through the blocking device without negative ions or the electric field. Upon applying negative ions and an electric field, the device started to block the mist (Fig. 6d and e). Furthermore, the mist was blocked at a position 10 cm from the device by the agarose collecting electrode, implying that agarose is superior to steel wool as a collecting electrode.

To quantify the performance of the collecting electrode, the fluorescent dye concentration in the water (Fig. 6a) was measured, which indicates the amount of the mist passed through the device depending on the device-height. The fluorescent dye concentration using the device with the agarose was smaller than that of the steel wool collecting electrode (Fig. 6f), indicating that agarose is superior to steel wool as a collecting electrode. This tendency is consistent with that shown in Fig. 6d and e. The fluorescent dye concentration increased depending on the device-height, indicating that blocking performance could not be sustained over a wide device-height range. In this experiment, we measured the current from the collecting electrode to the ground surface. The current decreased with the device-height as 20 to 30, 1 to 8, and 0 μA at 8, 16, and 20 to 50 cm, respectively. The decreasing current with device-height implied that an electric field with a wide device-height range did not guide negative ions, which causes a low blocking performance, as shown in Fig. 6f.

Figure 6g shows the blocking ratio; the blocking ratio was calculated based on the fluorescent dye concentration without negative ions and an electric field (Fig. 6f). The maximum blocking rate was 79% using the steel wool collecting electrode with a height of 8 cm, and the blocking ratio decreased with increasing device-height. Conversely, the maximum blocking rate was 89% using the agarose collecting electrode with a height of 16 cm, and the blocking ratio decreased over a device-height of 16 cm. The effective device-heights (blocking ratio > 0%) were 30 and 16 cm using agarose and steel wool as collecting electrodes, respectively. Therefore, Fig. 6 shows that agarose is superior to steel wool as a collecting electrode in terms of the effective device heigh.

### Blocking performance at different height positions

**Figure 7:**
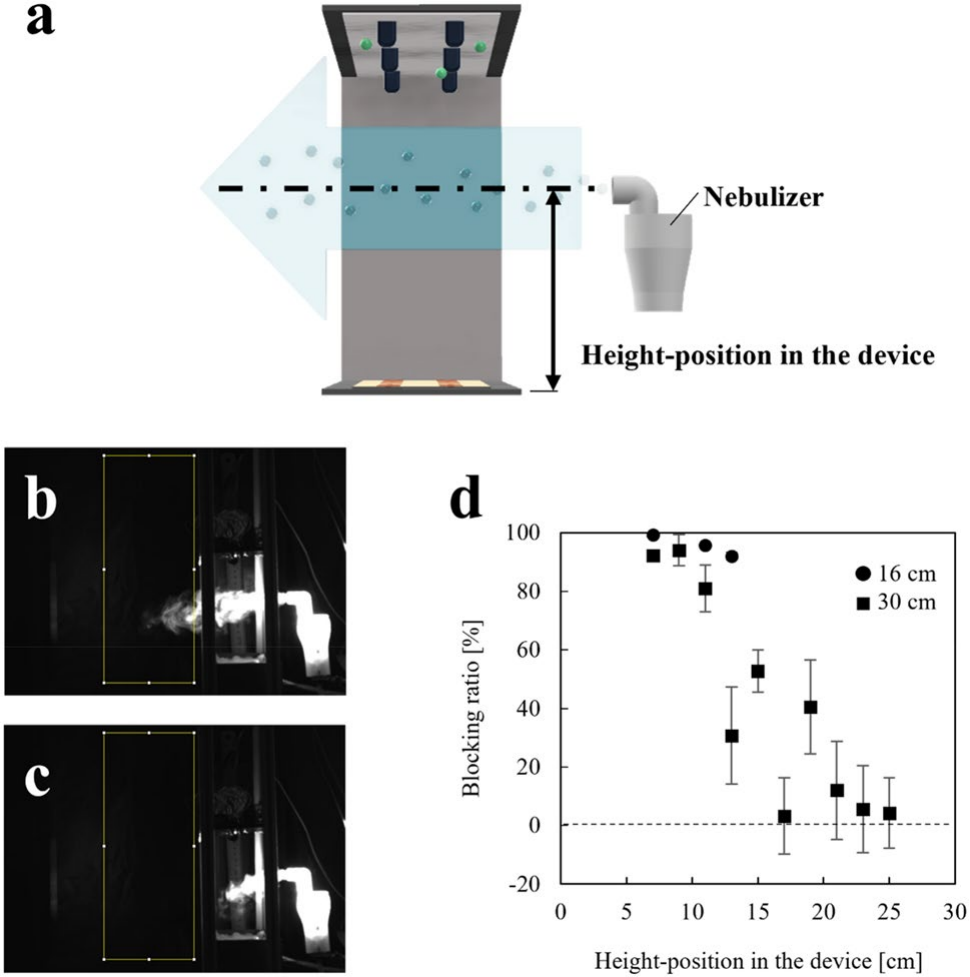
(**a**) Schematic of the experimental setup. (**b**,**c**) Device with a height of 16 cm and mist flow at a height-position of 9 cm. (**b**) Mist flow toward the device without negative ions and an electric field. (**c**) Mist flow toward the blocking device with negative ions and an electric field. (**d**) Blocking ratio of the device with heights of 16 and 30 cm.

The distribution of negative ions changed with the device–height (Fig. 5), and the blocking performance was also influenced by it (Fig. 6). Therefore, we predict that the blocking performance is related to the height-positions in the device (Fig. 7a). To evaluate the blocking performance at different height-positions in the device, the amount of mist that passed through the device was determined using the luminance intensity. The luminance intensity was measured in the area surrounded by the yellow frame shown in Fig. 7b and c. Figure 7d shows the blocking ratio calculated using the luminance intensity. The device with a height of 16 cm showed a gradual decrease in the blocking ratio; however a blocking ratio of 92% was maintained for height-position of 7 to 13 cm. By contrast, the device with a height of 30 cm showed a sharp decrease in the blocking ratio, suggesting that the blocking performance was higher around the collecting electrode than around the ground. The blocking ratio was under 60% for device with height-position of 13 to 25 cm. Figure 7d suggests that positioning the collecting electrode close to the droplets/aerosol is required to enhance the bocking performance against the droplets/aerosol.

### Transmission and reflection of light and sound

**Figure 8:**
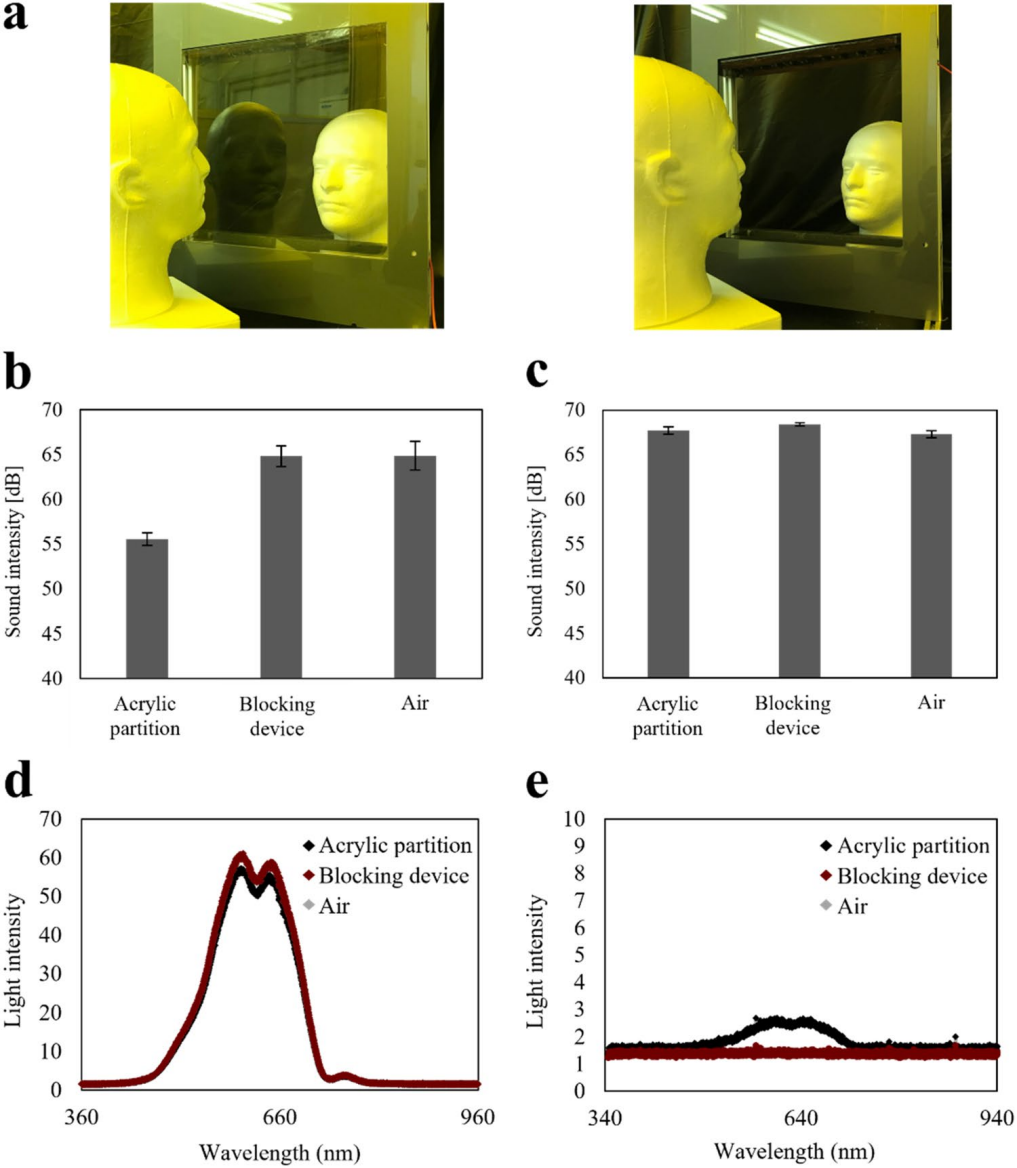
(**a**) Pictures demonstrating the device with the acryl partition and blocking device. (**b**) Transmitted sound intensity. (**c**) Reflected sound intensity. (**b**,**c**) Error bars indicate the deviation of three experiments. (**d**) Transmitted light intensity. (**e**) Reflected light intensity.

Partitioning using transparent materials interferes with light and sound, thereby disrupting communication. For example, as shown in Fig. 8a, an acryl partition reflects a doll's fac suggesting that it prevents communication. However, our device shows no light reflection, and the doll's face can be observed clearly. We evaluated the transmission and reflection of light and sound to confirm the acoustic and optical properties of our device. Figure 8b shows the sound transmission properties. The sound transmission of the device was as high as that of air (65 dB) in contrast to the acryl partition (56 dB), which reduces the sound level. In addition, Fig. 8c shows no significant difference in sound reflection. Therefore, Fig. 8b and c indicate that the negative ion and electric field in the device do not acoustically influence the sound. Figure 8d shows the light transmission spectra. The spectrum of the device overlapped with that of air, indicating no significant decrease in light due to the negative ions and electric field in the device. However, the acryl partition decreased light transmission (peak intensity of 57 at 603 nm) in contrast to the developed device (peak intensity of 61 at 603 nm). Subsequently, Fig. 8e shows the light reflection spectra. The spectrum of the device overlapped with that of air (peak intensity of 1.71 at 603 nm), indicating no significant reflection of the light by the negative ions and electric field in the device. Conversely, the spectrum of the acryl partition demonstrated reflection and a peak intensity of 2.7 at 603 nm. Figure 8b–e indicate that the negative ions and electric field in the device do not interfere with either sound or light.

### Experimental study using COVID-19 patients

**Figure 9:**
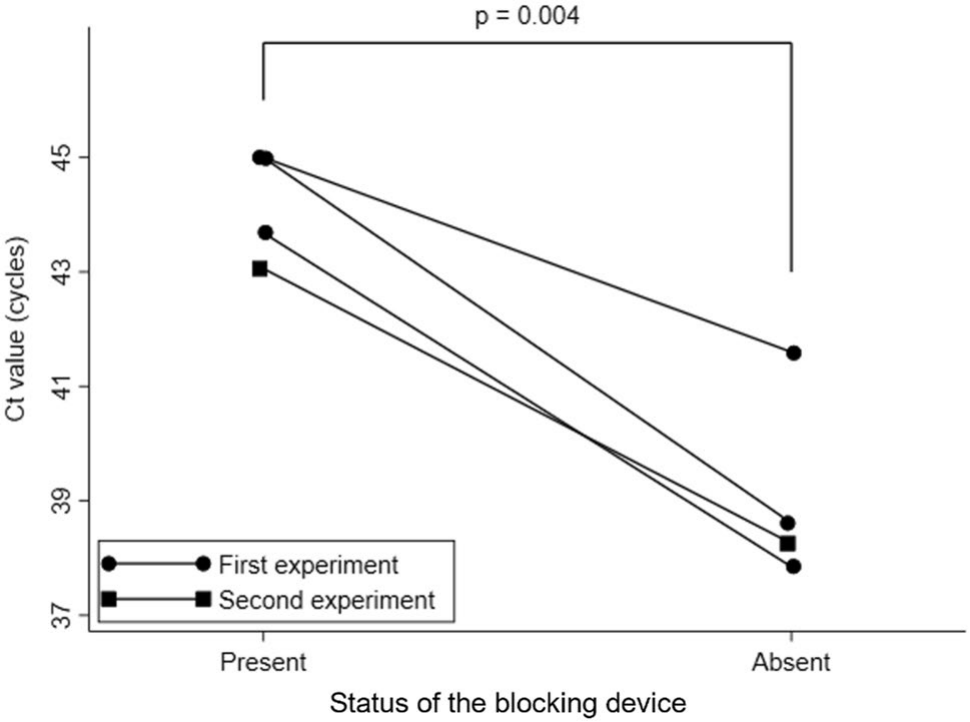
Ct value distribution of samples according to the status of the blocking device. The solid circles and rectangles represent samples from the first (N = 3) and second (N = 1) experiments, respectively. The symbols connected with a solid line indicate samples from the same participant. The P value was derived from a paired t-test.

The characteristics of the study participants are summarized in Table 1. The mean age was 49.7 (standard deviation (SD) = 15.1) years, and two participants were males. None of the participants had any underlying respiratory comorbidity. The mean Ct values of the saliva samples at admission and on the day before study participation were 27.0 (SD = 0.8) and 27.7 (SD = 3.1), respectively. Two participants conducted the first experiment, and one subject performed both the first and second experiments. The experimental results are shown in Fig. 9. Of the four pairs of samples, three pairs were obtained in the first experiment and one pair was from the second. The mean Ct values were 44.2 (SD = 0.98) with the blocking device and 39.1 (SD = 1.71) without the blocking device. The mean Ct value with the blocking device was significantly higher than that without the blocking device (P = 0.004).

**Table 1:** Characteristics of the study participants (N = 3).

	Mean (SD) or N (%)
Age (years)	49.7 (15.1)
Sex	
Male	2 (66.7)
Female	1 (33.3)
Underlying medical conditions	
Arrhythmia	1 (33.3)
None	2 (66.7)
Vaccination status	
Not at all	1 (33.3)
Twice	1 (33.3)
Missing	1 (33.3)
Pharmaceutical treatment	
Dexamethasone	1 (33.3)
Remdesivir	1 (33.3)
Sotrovimab	2 (66.7)
Duration from symptom onset or positive test result to admission (days)	2.7 (2.5)
Duration from admission to study participation (days)	2.3 (1.5)
Ct value at admission	27.0 (0.8)
Ct value on the day before study participation	27.7 (3.1)

*SD* standard deviation, *Ct* cycle threshold.

## Conclusion

In this study, a blocking device utilizing negative ions and an electric field was developed. The blocking device can guide negative ions toward the collecting electrode using an electric field, which is important for blocking charged droplets/aerosol. Comparing the blocking performances obtained using agarose and steel wool as the collecting electrode, agarose (with an effective device-height of between 8 and 30 cm) was superior to steel wool (with an effective device-height of between 8 and 16 cm). The device with a height of 16 cm using agarose blocked the mist by 89% compared to the device without negative ions and the electric field. The blocking performance depends on the height-position of the mist, and the performance deterioration of larger devices is related to the amount of mist passing through at high positions. Finally, the device demonstrated an effective blocking performance for aerosol containing the COVID-19 virus. Although the experimental study demonstrated the blocking capability of the blocking device, some limitations should be noted when interpreting the results. First, the sample size was small; therefore, the findings may be biased. Second, the experimental conditions were too simplistic to generalize the results to real-life scenarios. Future experimental studies with larger sample sizes that mimic real-life situations are warranted. Overall, the use of negative ions and electric field demonstrates the ability to block mist or aerosol containing the COVID-19 virus and transmit sound/light. This performance is beneficial as a sustainable countermeasure against infection during communication.

## Methods

### Electric field simulation

The CST Studio Suite (SIMULIA, U.S.) was used for the electric field analysis, and the finite integration technique was used for the calculations. The simulation model comprised a collecting electrode, ground, acrylic plate, and aluminum frame. The electrical constants of the components are listed in Table 2. The collecting electrode and ground were applied at + 15 and 0 kV, respectively.

**Table 2:** Electric constants of components for the simulation model.

Components	Relative permittivity	Conductivity [S/m]
Collecting electrode	1	6.9 × 10^–7^
Ground	1	5.8 × 10^–7^
Acrylic plate	3.2	–
Aluminum frame	1	3.6 × 10^–7^

### Effects of negative ions and electric field

Three ionizers were used to generate negative ions. A copper mesh was placed around the ionizers as a ground. An aluminum plate was used as the collecting electrode. The smoke motion was observed using a high-speed camera (CV-74, KATO KOKEN Co. Ltd., Japan). The shooting condition had a resolution of 640 × 480 pixels and frame rate of 170 fps.

### Effect of the electric field to guide the negative ions

The device shown in Fig. 1a was used. The structure was constructed using an aluminum flame (2 × 2 cm) and acrylic plate (3 mm thick, the breakdown voltage was 20 kV/m; KURARAY CO., LTD.). Eleven ionizers were placed to generate negative ions. An aluminum mesh was placed around the ionizers as a ground. Agarose (500 g) (as the collecting electrode material) was placed in a box (6 × 30 cm, 4 cm depth) on the underside of the blocking device. The agarose was prepared with 1 L of ultrapure water, 200 g of NaCl (FUJIFILM Wako Co., Ltd.), and 30 g of Agarose S (FUJIFILM Wako Co., Ltd.). The ion counter (COM-3200PRO II, Com Systems) was set at the center position of each area divided into 2 × 10 cm sections inside the device and 10 × 10 cm sections outside of the device to measure the negative ion concentration distribution. The negative ion concentration was measured every second for 1 min and the average value was plotted.

### Blocking performance of the blocking device

The blocking device (shown in Fig. 1a) was used with agarose and 30 g of steel wool (stainless steel wool, EIGHT CO-OPERATIVE BUYING CO., Ltd.) as the collecting electrode. The blocking device-height was adjusted between 8 and 50 cm. The nebulizer (NE-C28, OMRON Co.) was used to simulate aerosol released by an individual with a mild cough at a flow speed of 2 m/s^13^. The distribution of initial particle size was as follows: < 3 μm, 20%; 3 to 5 μm, 40%; > 5 to 8 μm, 40%, and the mass median diameter was 5.5 ± 0.2 μm^14^; notably, some mist particles gradually evaporate and change to aerosols^13^ (≤ 5 μm)^15^. The nebulizer was placed 20 cm from the blocking device, and the height of the nozzle was set at the center of the device-height. A saturated aqueous solution of the fluorescent dye uranine (FUJIFILM Wako Co. Ltd.) (4.1 mM) was sprayed horizontally toward the blocking device with a nebulizer for 10 min. The mist particles that passed through the blocking device were collected in a container filled with 400 ml of ultrapure water. The liquid sample in the container, including the mist particles attached to the container wall, was collected. The fluorescence intensity of the samples was measured using a multi-label plate reader (EnSpire 2300-00J, Perkin Elmer Co. Ltd.) at an excitation wavelength of 480 nm and emission wavelength of 512 nm. The concentration of fluorescent dye in the sample was calculated by referring to the calibration curve (Fig. S1). The mist flow was recorded using a high-speed camera.

### Blocking performance at different height positions

The blocking device (shown in Fig. 1a) was used, and the device-height was adjusted to 16 and 30 cm. The nebulizer was located at a distance of 5 cm from the blocking device. The height of the mist flow was varied, and the mist flow passing through the blocking device was captured. The mist flow containing the fluorescent dye was recorded with a high-speed camera, and the luminance was calculated using ImageJ (Rasband, National Institutes of Health, USA).

### Transmission and reflection of light and sound

The blocking device (shown in Fig. 1a) was used, and the device-height was adjusted to 30 cm. The acrylic partition was fabricated by attaching a 6-mm thick clear acrylic plate to the blocking device. To measure the sound transmission, the source of the sound and detector were symmetrically placed 20 cm from the blocking device. The sound source was a timer alarm. The sound detector was an iPhone 7 with an iOS platform app (Sound Level Analyzer Lite (iOS version 6.0.2))^16^. In addition, light transmission was evaluated atthe light source and detecter, which were placed at the same position of the light source and detector. The light source was an illuminator (Fiber Illuminator C-FI115, Nikon Co., Ltd.). A fluorescence spectrometer (USB4000-FL, Ocean Optics) was used as the light detector. To measure the specular reflection of the sound/light, the source and detector were placed 20 cm from the blocking device at 10°.

### Experimental study using COVID-19 patients

To demonstrate the efficacy of the developed blocking device, we conducted an experimental study using COVID-19 patients. The study participants were those who tested positive for SARS-CoV-2 using a real-time reverse transcription polymerase chain reaction (RT-PCR) test and were hospitalized at the Kuramochi Clinic Interpark^17^, Utsunomiya, Japan, in February and March 2022. The institution used to care exclusively for outpatients before the COVID-19 pandemic; inpatient beds were installed in March 2021 to deal with the increasing number of COVID-19 patients.

Two study configurations were implemented. First, the experimental room was equipped with two box-type air collectors, one was installed with an blocking device, while the other was not (Fig. S2a). Air samples were collected using Aerosolense Air Sampler (Thermo Fisher Scientific, MA, USA) at 200 L/min. Air samplers were allocated for ambient air to flow into the boxes through the air inlets and discharged out of the boxes from the air outlets. Considering the box with the blocking device, the air entering the box passed through the blocking device before collection by the air sampler (Fig. S2b), whereas air entering the box without the blocking device was directly trapped by the air sampler (Fig. S2c). The participants stayed in the experimental rooms for 2 h. To ensure that participants excreted droplets and aerosol, participants read passages derived from old tales (e.g., The Blue Bird) for 5 min per hour to promote spit diffusion. Ambient air was collected in parallel using two boxes, and a pair of samples was obtained from each participant. The second experiment was similar to a real-life scenario. The boxes were removed, and the built-in ventilators were turned on. The experiment was arranged in two ways: one setup was equipped with the blocking device and air sampler in front of the built-in ventilator (Fig. S2d), and the other with an air sampler and a built-in ventilator (Fig. S2e). These conditions were conducted serially for the same participant for 2 h each. The participants again read old tales for 5 min per hour. In this experiment, there was no box surrounding the blocking device and air sampler, implying that not all the air collected by the air sampler passed through the blocking device (Fig. S2f). If the blocking device was not applied, ambient air was directly collected using an air sampler (Fig. S2g).

Three participants were recruited, none of whom experienced hypoxia. We obtained three pairs of samples from the first experiment and one pair of samples from the second experiment (one participant conducted both experiments). SARS-CoV-2 testing was conducted via real-time RT-PCR with a CFX96 Real-Time System (Bio-Rad, Hercules, CA, USA) and a SARS-CoV-2 detection kit comprising primers and probes (RC30JW, TaKaRa, Japan). Real-time RT-PCR was conducted under the reaction conditions described in the manual of the National Institute of Infectious Diseases in Japan^18^. If the viral load was below the detection limit, the corresponding sample was assigned a Ct value of 45, the maximum cycle number according to the real-time RT-PCR protocol^18^. The difference between the mean Ct values of the samples from the two groups (with or without the blocking device) was tested using a two-tailed paired t-test, in which the significance level was set at 0.05. Written informed consent was obtained from all participants. The experimental study was approved by the Ethics Committee at Kuramochi Clinic Interpark (IPK-0002), and performed in accordance with the Declaration of Helsinki and the Japan's Ethical Guidelines for Medical and Biological Research Involving Human Subjects established by the Ministry of Education, Culture, Sports, Science and Technology, the Ministry of Health, Labour and Welfare, and the Ministry of Economy, Trade and Industry.

### Supplementary Information

Supplementary Figures.

Supplementary Information 2.

## Data Availability

All data needed to evaluate the conclusions in the paper are present in the paper and/or the Supplementary Materials. Additional data related to this paper may be requested from the authors.
