# Supplementary material for: Applying negative ions and an electric field to countermeasure droplets/aerosol transmission without hindering communication
Source: Sci Rep. 2023 Aug 26;13:13965. doi: 10.1038/s41598-023-40303-5 (PMC10460439; doi:10.1038/s41598-023-40303-5)
Supplement: Supplementary file 1 — Supplementary Figures. [file 41598_2023_40303_MOESM1_ESM.docx]

**Supporting Information**


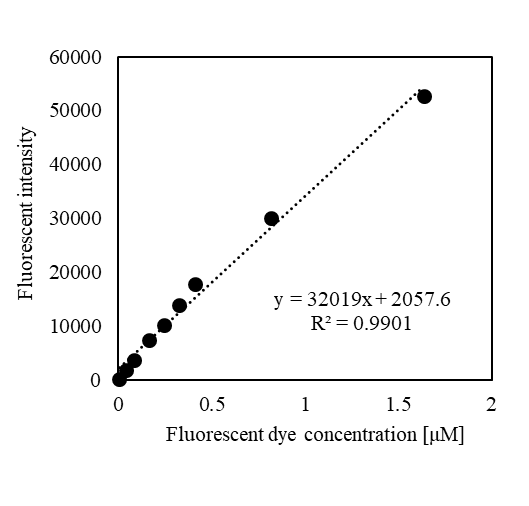


Fig. S1. Calibration curve indicating the relationship between fluorescent dye concentration and intensity.


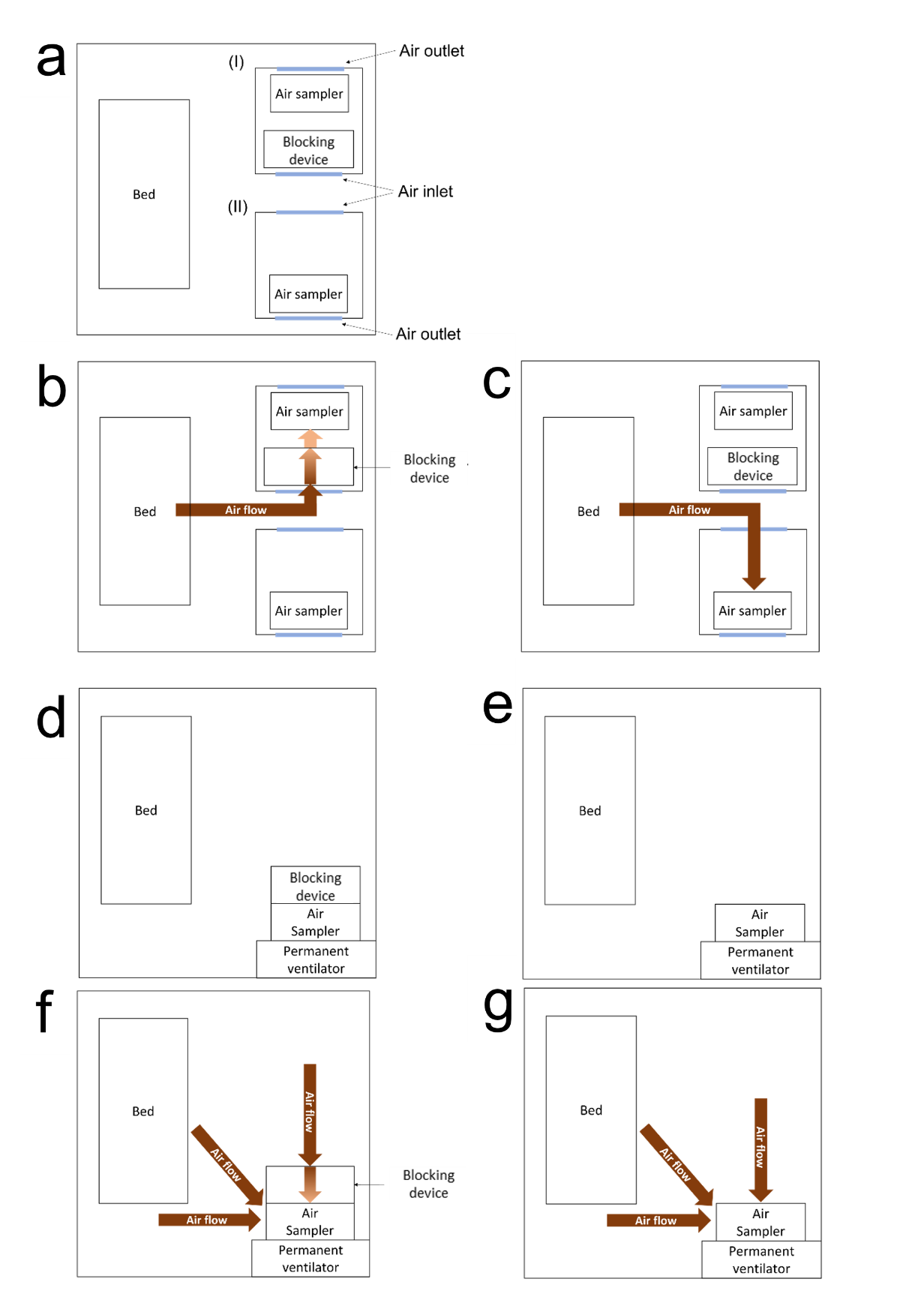


Fig. S2. Schematics of the experimental configuration. For the first experiment, the experiment room was equipped with a bed for the participant and two box-type air collectors **a**: one containing an air sampler together with the blocking device in front of the air sampler (I) and the other equipped with an air sampler only (II). Samples were collected in the two air samplers parallelly for the same participant. Air samplers were arranged so that ambient air flowed into the box through the air inlet and was discharged out of the box through the air outlet. This configuration made sure that air flowing into a box with the blocking device passed through the blocking device prior to collection by the air sampler **b**, while air coming into a box without the blocking device was directly collected by the air sampler **c**. For the second experiment, in which we assumed a more realistic condition, we removed the boxes and the permanent ventilators were turned on. The experiment was arranged in two ways and conducted serially for the same participant: one with an air sampler and the blocking device placed in front of the permanent ventilator **d**, and the other with an air sampler and the permanent ventilator only **e**. In this configuration, even if the blocking device was applied, air collected in the air sampler did not necessarily pass through the blocking device: the air sampler collected both air that passed and did not pass through the blocking device **f**. If the blocking device was not applied, the air was directly collected in the air sample **g**.


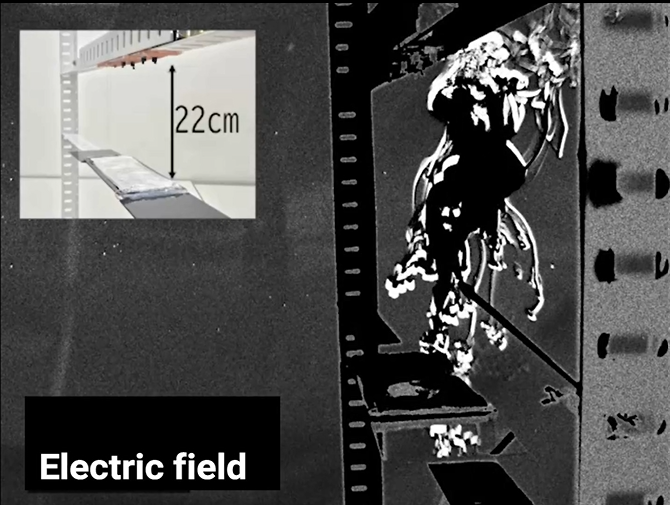


Video S1. The effects of negative ions and the electric field on the smoke.


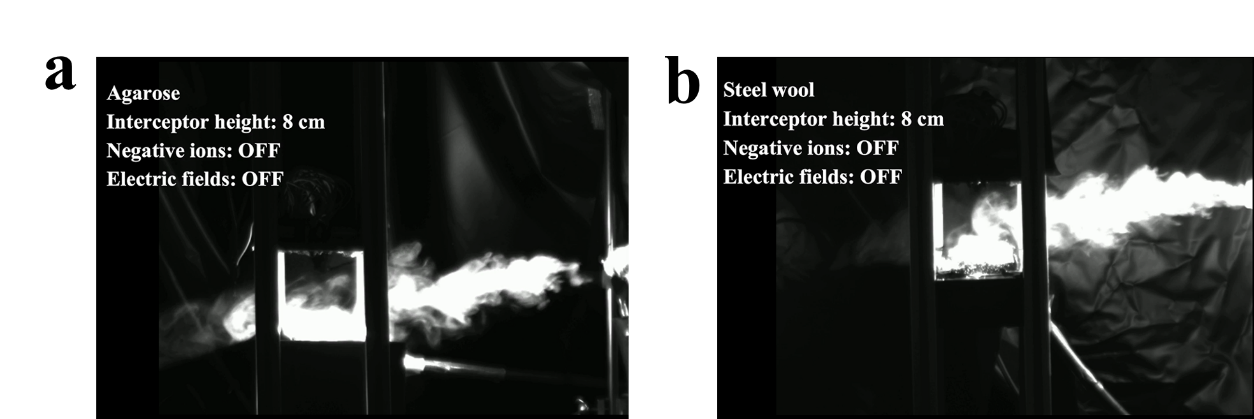


Video S2. The effect of the blocking device with different heights on the mist. **a** Agarose was used as the collecting electrode. **b** Steel wool was used as the collecting electrode.
